# Adipose tissue lipolysis and remodeling during the transition period of dairy cows

**DOI:** 10.1186/s40104-017-0174-4

**Published:** 2017-05-05

**Authors:** G. Andres Contreras, Clarissa Strieder-Barboza, William Raphael

**Affiliations:** Department of Large Animal Clinical Sciences, College of Veterinary Medicine, Michigan State University, East Lansing, MI 48824 USA

**Keywords:** Adipose tissue macrophages, Adipose tissue remodeling, Lipolysis, Transition dairy cows

## Abstract

Elevated concentrations of plasma fatty acids in transition dairy cows are significantly associated with increased disease susceptibility and poor lactation performance. The main source of plasma fatty acids throughout the transition period is lipolysis from adipose tissue depots. During this time, plasma fatty acids serve as a source of calories mitigating the negative energy balance prompted by copious milk synthesis and limited dry matter intake. Past research has demonstrated that lipolysis in the adipose organ is a complex process that includes not only the activation of lipolytic pathways in response to neural, hormonal, or paracrine stimuli, but also important changes in the structure and cellular distribution of the tissue in a process known as adipose tissue remodeling. This process involves an inflammatory response with immune cell migration, proliferation of the cellular components of the stromal vascular fraction, and changes in the extracellular matrix. This review summarizes current knowledge on lipolysis in dairy cattle, expands on the new field of adipose tissue remodeling, and discusses how these biological processes affect transition cow health and productivity.

## Background

### Adipose tissues, the energy warehouse during the transition period

Adipose tissue (AT) functions as the major body of energy reserve in mammals. During positive energy balance, AT stores energy surplus as fatty acids (FA) incorporated into triacylglycerols (TAG) in a process known as lipogenesis. In de novo lipogenesis, also defined as de novo FA synthesis, FA are derived from carbohydrate and amino acid carbons through acetylCoA. Additionally, AT directly esterifies circulating free FA from dietary or other metabolic origins (e.g. hepatic lipolysis) [1, 2]. The majority of FA incorporated into TAG come from de novo FA synthesis, however, circulating FA may supply up to 50% of FA, especially during early lactation [2, 3]. When energy is limited, the AT releases FA from TAG through lipolysis [4]. Due to its unique lipogenic and lipolytic responses, AT is the only organ capable of extensive growth and reduction at any stage of life [5].

The transition period of dairy cows is defined as the time when the physiological state changes from gestational non-lactating to non-gestational lactating and occurs from 3 wks before through 3 wks after parturition [4]. During the transition period, hormonal changes associated with parturition and the initiation of lactogenesis, including the reduction in progesterone and the surge of prolactin and growth hormone (GH), favor lipolysis over lipogenesis independently of energy balance status [6]. Lipolysis rate is further exacerbated by intense energy requirements, especially glucose, associated with the rapid fetal growth, parturition, the onset of lactation, and reduced dry matter intake, leading to negative energy balance (NEB) [7, 8]. Intense and protracted lipolysis during the transition period leads to AT size reduction and alterations in the secretion pattern of adipokines that favor the use of FA as an energy source for bodily maintenance and glucose for fetal growth and lactation [8, 9]. In addition, lipolysis induces a remodeling process within the adipose organ that is characterized by an inflammatory response, immune cell infiltration, cellular proliferation, and extracellular matrix (ECM) changes [10, 11]. During the transition period, lipolysis-induced AT remodeling coincides with a period of reduced insulin sensitivity in myocytes, hepatocytes, and adipocytes that redirects energy for milk production in the mammary gland [12]. As lactation progresses, bovine adipocytes become more responsive to insulin, resulting in reduced lipolysis rates and enhanced lipogenesis [2]. AT insulin resistance during the transition period ensures a healthy and productive lactation when moderated; however, when intense and protracted, it predisposes cows to inflammatory and metabolic diseases by limiting AT capacity for energy buffering and contributing to chronically increased plasma FA [12, 13]. In this review, we will discuss the different aspects of adipose tissue lipolysis and remodeling during the transition period, and their impact on transition cow health and lactation performance.

## Lipolysis

A key characteristic of the transition period is intense lipolysis. In general, lipolysis can be broadly divided into two categories: basal and demand lipolysis [14]. In humans and rodents, the rate of basal lipolysis is determined by adipocyte size and TAG content [10, 15]. Similarly, in dairy cows, basal lipolysis rate is positively associated with adipocyte size and increases steadily throughout lactation [16–19]. In contrast, demand lipolysis is regulated hormonally in response to energy demands. Independent of the type of lipolysis, TAG within the adipocyte lipid droplet are broken down by the action of three different lipases: adipose triglyceride lipase (ATGL), hormone sensitive lipase (HSL), and monoglyceride lipase (MGL). However, HSL and MGL have a larger role in demand lipolysis than basal lipolysis. The actions of these lipolytic enzymes, explained in subsequent paragraphs, are regulated by different co-activator proteins that also exhibit changes in expression during the transition period.

### Lipases

The primary regulator of basal lipolysis in monogastric animals is ATGL (Table 1). This lipase acts exclusively on TAG. ATGL is activated by CGI-58, also known as α/β hydrolase domain-containing protein 5 [20]. Upon lipolytic stimulus and consequent phosphorylation of lipid droplet associated protein perilipin 1 (PLIN1), CGI-58 is released into the cytoplasm and activates ATGL by direct protein-to-protein interaction [20]. In transition dairy cows, ATGL-encoding gene, *PNPLA2*, is downregulated during the last week of gestation and the first week of lactation compared to the dry period and mid lactation [21, 22]. Similar to the *PNPLA2* expression pattern, ATGL abundance remains decreased during the transition period in comparison to the content observed at 4 wks pre-calving [21]. CGI-58 gene expression is also reduced after calving; however, its protein abundance remains unchanged as the transition period progresses [21, 23]. The patterns of gene expression and the protein abundance of ATGL, as well as its coactivator CGI-58, indicate that basal lipolysis is maintained at a steady rate, suggesting that demand lipolysis is the main source of plasma FA during the transition period.

**Table 1:** Major components of lipolysis pathways in adipose tissue (AT) of dairy cows

Protein(s)	Encoding gene(s)	Activation stimulus	Function(s)	Transition period dynamics
Catecholamines (adrenaline and noradrenaline)		Metabolic stress [125]	- Bind to βAR on the adipocyte cell surface and initiate the lipolytic cascade [33]	- Plasma adrenaline and noradrenaline: - Increase during the dry period and immediately before calving - Decrease slightly after calving until 60 d in lactation [62]
Adipocyte β-adrenergic receptors (βAR), types: β_1_R, β_2_R, and β_3_R.	*B1AR* *B2AR* *B3AR*	Catecholamine binding	- Activate adenylyl cyclases that convert ATP to cAMP, and induce PKA activation that, in turn, phosphorylates PLIN1 initiating the lipolytic cascade - βAR are increased in abdominal adipose and during increased rate of lipolysis [33]	- βAR increase during first month after parturition and are positively associated with milk production [27] - B2AR decreases immediately after parturition and increases after peak lactation [27]
Perilipin-1	*PLIN1*	PKA	- Protects the lipid droplet from hydrolytic activity of HSL	- Decreases during first 3 wks following parturition compared to other periods of the lactation cycle [21]
α/β hydrolase domain-containing protein 5 (ABHD5/CGI-58)	*ABHD5*	Phosphorylation of PLIN1	- Increases the activity of ATGL and provides increased DAG substrate for PKA-activated HSL	- Decreased gene expression after calving - Protein abundance remains unchanged through the transition period [21]
Adipose tissue triglyceride lipase (ATGL)	*PNPLA2*	ABHD5/CGI-58	- Hydrolysis of TAG ^c^	- Decreased gene expression during the last week of gestation and first week of lactation compared to the dry period and mid lactation - Lower protein abundance around parturition compared to the dry period [21, 22]
Hormone sensitive lipase (HSL)	*LIPE*	Activation of PKA	- Hydrolyzes DAG ^b^, releasing free fatty acid and MAG ^a^ - Activity varies depending on anatomical location of AT	- Decreased gene expression during the first 3 wks following parturition compared to the dry period [23] - Protein content unchanged through the transition period [21, 123]
Fatty acid-binding protein (aP2/FABP4)	*FABP4*	Association with HSL	- Transports fatty acids to the plasma membrane	- Increased gene expression during induced milk fat depression [126]
Monoglyceride lipase (MGL)	*MGll*	DAG ^b^ hydrolysis by HSL	- Hydrolyzes MAG ^a^, releasing glycerol	- Decreased gene expression during the first 3 wks of lactation [27] - Protein content currently unknown in cattle

^a^ MAG: Monoglycerol

^b^ DAG: Diglycerol

^c^ TAG: Triglycerol

HSL is the rate-limiting factor for demand lipolysis ([24, 25], Table 1). This lipase hydrolyses several lipid substrates including TAG, diglycerides, monoglycerides, and cholesterol esters [14]. Upon activation by protein kinase A (PKA), HSL associates with fatty acid binding protein 4 (FABP4) to form a complex that localizes on the lipid droplet. The expression of *LIPE*, the gene encoding HSL, is reduced during the first 3 wks after parturition compared to the dry period [16, 23]. The protein content of HSL remains unchanged throughout the transition period. However, the rate of phosphorylation at different active sites, and therefore its lipolytic activity, is increased during the first 3 wks after calving [21]. In rodents, HSL requires phosphorylation at Ser^563^, Ser^659^, and Ser^660^ by PKA or protein kinase G (PKG) to initiate TAG hydrolysis. Although HSL serine residue activation sites have not been determined in transition cattle, increased phosphorylation at Ser^563^ and Ser^660^ was reported postpartum and during feed restriction protocols [21, 26]. Notably, HSL activity varies depending on anatomical location of adipose tissues. Locher et al. [26] reported higher phosphorylation rates at Ser^563^ and Ser^660^ in retroperitoneal AT from dairy cows at 21 d of lactation compared to subcutaneous depots. In the same study [26], phosphorylation rates, especially those at Ser^563^, were associated with higher lipolytic activity.

A complete activation of the lipolytic process requires not only the activation of ATGL and HSL, but also the phosphorylation of PLIN1. This protein protects the lipid droplet from the lipolytic activity of HSL. PLIN1 is phosphorylated by PKA to allow HSL interaction with TAG. In dairy cows, PLIN1 phosphorylation is increased during the first 3 wks after parturition, compared to other periods of the lactation cycle [21]. In transition dairy cows, lipolysis is modulated by post-transcriptional and allosteric changes in the HSL hydrolase pathway. Although differences in lipolysis rate are reported depending on the adipose tissues’ anatomical site, it is currently unknown if these changes may impact disease susceptibility or lactation efficiency.

The lipolytic pathway is completed by MGL, which acts exclusively on monoglycerides (Table 1). In dairy cows, the gene transcription patterns of *MGll*, which encodes MGL, follow those of *PNPLA2* and *LIPE*, with lower expression during the first 3 wks of lactation [27]. The dynamics of MGL protein expression are currently unknown in cattle, and it is uncertain if changes in its activity may affect lipolysis rates, as HSL is very active on monoglycerides and MGL hydrolyses these lipid species exclusively.

### Regulation of lipolysis during the transition period

In rodents and humans, catecholamines and natriuretic peptides are the most important activators of lipolysis in AT [28]. In the same species, both insulin and catecholamines (acting through alpha adrenergic receptors) are negative regulators of TAG hydrolysis (Table 1). In cattle, the role of catecholamines and insulin in lipolysis modulation is well-documented; however, it is unknown if natriuretic peptides significantly modify lipolytic pathways.

Catecholamines bind to β-adrenergic receptors (βAR) on the adipocyte cell surface, activating adenylyl cyclases that convert ATP to cAMP (Table 1). Accumulation of cAMP induces PKA activation, which in turn phosphorylates PLIN1 and initiates the lipolytic cascade [29]. There are 3 major types of βAR: β_1_, β_2_, and β_3_. In dairy cows, the expression of all βAR-encoding genes was initially reported in mammary gland [30], and,more recently, in subcutaneous AT [16, 27] Only β_1_ and β_2_ were demonstrated to have lipolytic responses in the AT of cows, and stimulation of the latter was directly associated with increases in plasma FA during NEB [31]. Similar to the gene expression of lipolytic enzymes, the transcription of *B2AR* (encoding β_2_AR) is downregulated immediately after parturition and rises again only after peak lactation [27]. A classical study by Jaster and Wegner [32] revealed that the activity and responses to adrenergic stimuli of the βAR in the subcutaneous AT was increased during the first month after parturition compared to the dry period. The dynamics of AT βAR response during the dry and lactation periods were completely characterized by McNamara and Hillers [17] who demonstrated that lipolytic responses to epinephrine peak at 1 month postpartum and remain elevated 6 months into lactation compared to 1 month pre calving. In the same study, lipolytic responses to norepinephrine stimulation remained higher during lactation compared to the dry period. Strikingly, the lipolytic response triggered by βAR stimulation in early lactation is increased in proportion to milk production [27]. Although not described in dairy cows, βAR density and the lipolytic activity in adipocytes after adrenergic stimulation may vary depending on the anatomical location of the adipose depot. In sheep, there are more βAR present in omental adipocytes, compared to subcutaneous adipocytes, after 2 wks of lactation [6]. The impact of the higher content of βAR in abdominal AT is reflected in the higher rate of lipolysis from these depots during NEB states related to lactation. In fact, the FA profiles of the plasma NEFA fraction in cows with intense lipolysis, such as those with displaced abomasum, are remarkably similar to the FA composition of visceral adipose depots, especially in the content of saturated and monounsaturated FA [33].

Adipocytes are one of the most highly insulin-responsive cell types [34]. In adipocytes, insulin stimulates glucose transport and lipogenesis, promotes the uptake of FA from systemic circulation, and inhibits lipolysis [34, 35]. Insulin binds and activates the insulin receptor tyrosine kinase, resulting in the phosphorylation of insulin receptor substrates 1 and 2 (IRS-1/IRS-2) [36]. These events are followed by activation of phosphatidylinositol 3-kinase (PI3K), phophoinositide-dependent kinase1 (PDK1), and protein kinase B (AKT), which mediate insulin metabolic and mitogenic effects, including glucose uptake through glucose transporters (e.g. GLUT4) [35]. Insulin suppresses lipolysis through the activation of AKT, which results in the inhibition of downstream protein kinase A (PKA) and reduces PKA phosphorylation of PLIN1 [37]. In dairy cows, decreased circulating insulin and AKT phosphorylation in the liver and AT stimulate gluconeogenesis and lipolysis during the transition period and early lactation [8, 12, 38].

During the transition period, changes in the secretion patterns of GH, angiopoietin-like 4 (ANGPTL4), and prolactin further modulate lipolysis. GH activates lipolysis and reduces insulin sensitivity in mammalian AT [39]. In lactating dairy cattle, GH administration augments 2 to 6-fold AT response to adrenergic stimulation [40]. In vitro experiments with AT from lactating animals indicate that GH reduces adipocyte sensitivity to anti-lipolytic molecules, such as adenosine, that inhibit the activity of adenylyl cyclase [41]. Thus, compared to non-lactating cows 4 to 8 wks prior to parturition, cows in the transition period have elevated circulating GH which enhances lipolysis by increasing adipocyte responses to adrenergic stimuli and reducing the inhibitory effects on sympathetic activity through adenosine.

ANGPTL4, also known as fasting-induced adipose factor, is an adipokine secreted during NEB that inhibits adipocyte uptake of FA for esterification [42]. ANGPTL4 plays a key role in enhancing lipolysis in adipocytes, especially during catecholamine stimulation, by increasing cAMP and enhancing the phosphorylation of PKA [43]. ANGPTL4 transcription and synthesis is elevated in response to glucocorticoids [43], possibly indicating that this adipokine facilitates lipolytic response during stress periods, such as parturition. In dairy cows, Koltes and Spurlock [44] described the dynamics of *ANGPTLl4* gene expression following lipolysis induction with GH, as well as during NEB states, including the transition period and during feed restriction. *ANGPTL4* transcription was inversely associated with the degree of NEB in all three models of limited energy status. Although ANGPTL4 protein content in AT from transition cows has not been determined, it is expected to reflect *ANGPTL4* transcription, similar to what has been observed in rodents [45]. Thus, in dairy cattle, ANGPTL4 supports lipolysis during NEB stages, including the transition period.

In addition to supporting lactation, prolactin promotes adipocyte lipolysis in vivo. Furthermore, continuous exposure of fat cells to this pituitary hormone have been shown to reduce AT size in rabbits [46]. However, it is unknown if prolactin exerts the same effect in bovine adipocytes, and its effect on modulating lipolysis intensity during the transition period remains to be elucidated.

## Adipokines

The role of AT is now recognized as a major regulator of systemic metabolism which extends beyond energy buffering [47]. This function is accomplished by secreting specialized proteins that exert autocrine, paracrine, and endocrine functions. These proteins are termed adipokines and are produced by the cellular components of AT, such as adipocytes and cells of the stromal vascular fraction (SVF), including immune, vascular, and adipocyte progenitor cells. Despite the ever-expanding list of adipokines, which now accounts for over 300 secretory products [48], few have been studied in dairy cattle (Table 2). Among these signaling molecules, adiponectin and leptin are almost exclusively secreted by AT; while others, such as resistin and retinol binding protein 4, are also produced in the liver. Similarly, interleukin-6 (IL-6) and tumor necrosis factor alpha (TNFα) are not only produced by adipocytes, but also resident immune cells in AT (e.g. macrophages, lymphocytes, polymorphonuclear cells). Recent research provides evidence that the dynamics of adipokine secretion during the transition period drive the homeorhetic potential of dairy cows by redirecting glucose to the mammary gland, increasing FA flow to the liver, and modulating energy intake [49].

**Table 2:** Adipokine expression dynamics and roles in modulating adipose tissue (AT) lipolysis in transition dairy cows

Protein	Encoding gene	Function(s)	Transition period dynamics
Angiopoietin-like 4 (ANGPTL4)	*ANGPTL4*	- Inhibits LPL activity and adipocyte fatty acid (FA) uptake [42] - Increases cAMP production and enhances PKA phosphorylation through the integration of catecholamine and corticoid signaling [43] - Regulated by PPARs [127]	- Gene expression inversely associated with NEB intensity during transition period [44]
Adiponectin (Acrp30)	*ADIPOQ*	- Improves insulin sensitivity and promotes lipogenesis in adipocytes [9] - Enhances FA β-oxidation in myocytes and hepatocytes [9] - Promotes anti-inflammatory and resolving phenotypes in immune cells [54]	- Decreased gene expression immediately after calving compared to dry period - Plasma concentration peaks between 40–70 d of lactation [53, 54] - Decreased AT expression of its receptors (adipoR1 and adipoR2) during the first 3 wks after calving compared to dry period, increasing steadily to peak around 100 d of lactation [55, 56]
Leptin	*OB*	- Decreases lipogenesis and increases lipolysis and FA oxidation [59] - Reduction in leptin transcription and secretion during the transition period may promote a rapid return to normal DMI [62]	- Leptinemia peaks during the dry period and decreases during the first week following calving [60, 61] - Increased AT gene expression during the dry period contrasted with minimal transcription immediately following parturition [60]
Resistin	*RETN*	- Increases lipolytic rate and the transcription of ATGL and HSL in AT explants from transition cows [66]	- Increased in plasma after calving, returning to pre-partum concentration by 6 wks into lactation [66, 67] - AT transcription and secretion dynamics are similar to that of plasma resistin [66]
Retinol binding protein 4 (RBP4)	RBP4	- Serves as a carrier of retinol [68] - Impairs glucose uptake by adipocytes and hepatocytes by suppressing insulin signaling pathways [69] - Inhibits adipogenesis [70]	- Decreased circulating RBP4 during the first day after parturition followed by return to pre-calving levels by second week of lactation [71, 72]

Adiponectin, also known as ACRP30, is secreted primarily by adipocytes, but is also expressed by cardiomyocytes and skeletal muscle [50]. Adiponectin improves insulin sensitivity and lipogenesis in adipocytes, and FA β-oxidation in myocytes and hepatocytes [9]. These effects are exerted by the activation of its receptors (adipoR1 and adipoR2), which are expressed in liver, AT, and skeletal muscle [51]. In monogastric animals, circulating adiponectin is present in 3 major oligomeric forms (low-molecular weight (LMW), middle molecular weight (MMW), high molecular weight (HMW) and as globular adiponectin [51]. In dairy cows, circulating adiponectin is composed mainly of high molecular weight complexes and its distribution is not affected by stage of lactation [49, 52]. Circulating adiponectin reaches its nadir immediately after calving, then peaks between 40 and 70 d into lactation [53, 54]. Remarkably, concentrations of circulating adiponectin are inversely associated with plasma FA, the main lipolysis biomarker in dairy cows [54]. Similarly, AT expression of genes encoding adipoR1 and adipoR2 is downregulated during the first 3 weeks after calving and then increases steadily to peak at around 100 DIM [55, 56]. The dynamics of the expression and secretion of adiponectin and its receptors may indicate that this adipokine acts as an autocrine, paracrine, and endocrine modulator of the homeorhetic adaptations of AT during the transition period. However, the extent to which adiponectin affects glucose partitioning to the mammary gland in early lactation is currently unknown.

Leptin was one of the first adipokines characterized in mammals. Unlike adiponectin, leptin is expressed and secreted not only by adipocytes, but also by myocytes and mammary gland and gastric mucosa epithelial cells [57]. In dairy cows and other ruminants, the leptin encoding gene (*OB*) is expressed ubiquitously, including in the rumen, abomasum, duodenum, mammary gland, skeletal muscle, pituitary gland, and AT (reviewed in [58]). Similar to adiponectin, leptin acts in an autocrine, paracrine, and endocrine manner to modulate food intake and energy expenditure. This anorexigenic adipokine reduces lipogenesis and increases lipolysis and FA oxidation [59]. In dairy cows, plasma leptin concentration peaks during the dry period and decreases drastically in the first week after calving [60, 61]. In AT, *OB* expression reflects leptinemia dynamics, with the highest expression in the dry period and minimal transcription immediately following parturition [60]. Reduction in leptin transcription and secretion during the transition period may promote a rapid return to normal DMI; however, it is presently unclear if, in over-conditioned cows, hyperleptinemia enhances the onset of pre-calving lipolysis and losses of AT reserves [62, 63].

Resistin is an adipokine secreted by adipocytes and macrophages [64]. AT transcription and secretion levels of resistin are linked to the development of insulin resistance in humans, as well as in rodent models of obesity and metabolic syndrome [65]. In transition dairy cows, plasma concentration of resistin peaks during the first week after calving and returns to pre-partum levels after 6 wks of lactation [66, 67]. Plasma resistin is positively associated with plasma NEFA and negatively correlated with milk production [66, 67]. AT resistin transcription and secretion dynamics are similar to that of plasma resistin, with higher expression during the first week post-calving compared to other stages of lactation and the dry period [66]. Remarkably, resistin was associated with higher rate of lipolysis, as well as enhanced transcription of ATGL and HSL, in AT explants from transition cows [66].

RBP4 is a lipocalin transport protein secreted by adipocytes and hepatocytes that serves as a carrier of retinol [68]. Like resistin, RBP4 impairs glucose uptake by adipocytes and hepatocytes by suppressing insulin signaling pathways [69]. Enhanced expression of RBP4 inhibits adipogenesis, thus impairing the FA buffering capacity of AT during obesity or periods of high lipolytic rate [70]. In dairy cows, plasma RBP4 declines sharply after parturition and returns to pre-calving levels by the second week of lactation [71, 72]. In the AT of transition cows, *RBP4* transcription was detected and found to be positively associated with TNFα secretion [72].

## Adipose tissue remodeling

AT lipolysis not only involves the release of FA, neutral lipids, and glycerol, but also induces a remodeling process of the organ [10, 73]. This remodeling is characterized by an inflammatory response with immune cell migration, proliferation of the cellular components of the SVF, and changes in ECM of AT [5]. Intense research during the past two decades has characterized AT remodeling in human diseases that exhibit high AT lipolysis rates, such as obesity [5, 74], metabolic syndrome [75], and lipodystrophy [76]. More recently, different aspects of AT remodeling in dairy cows during the transition period and metabolic disease were evaluated. We summarize these reports from cattle studies below in the context of the current understanding of adipose tissue remodeling in humans and rodent models.

### Immune cell infiltration

Studies in multiple animal species and humans show dynamic populations of immune cells located within the SVF of AT [77–79]. Cells of the innate and adaptive immune systems, such as macrophages, mast cells, lymphocytes, neutrophils, eosinophils, dendritic cells, and NK, exhibit active AT trafficking during health and disease in rodents and humans [80], as well as in dairy cows during the transition period and late lactation [81, 82]

Macrophages are the most predominant cell type in monogastric and ruminant SVF [77, 79]. Adipose tissue macrophages (ATM) are a key component of the inflammatory response during lipolysis. The specific inflammatory phenotype of ATM has been broadly classified in classical ATM (M1), which have active pro-inflammatory responses, and alternative phenotype ATM (M2), which promote inflammation resolution. In dairy cows, ATM infiltration occurs as a response to intense lipolysis. In cases of displaced abomasum in transition dairy cows, macrophages exhibiting a M1 phenotype accumulate in aggregates within omental and subcutaneous depots [81]. During induced NEB in late-lactation cows, lipolysis induced ATM infiltration into subcutaneous and visceral AT, yet no phenotypic change was observed [82].

The role of ATM during lipolysis is to remove lipolytic products such as FA, diglycerides, and monoglycerides that, in excess, cause lipotoxicity in AT [83]. ATM are also involved in the recruitment of new adipocyte progenitors by secreting chemotactic proteins such as osteopontin [83]. The transcription of this latter protein was shown to be upregulated in AT of lactating dairy goats during feed deprivation [84]. The ATM phenotype is plastic in response to the milieu of adipocyte-derived molecules, such as adipokines and FA [79]. During lipolysis, ATM bind to saturated FA at toll-like receptors (TLR), resulting in the activation of NF-κB transcription factor pathways and inflammatory gene transcription, leading to M1 polarization [85–87]. ATM secretion of cytokines such as TNFα and interleukins 1β and 6 during lipolysis mediates local and systemic inflammation,6 [79, 88]. Interestingly, macrophage infiltration into AT enhances lipolysis, creating a vicious cycle that connects lipolysis, ATM infiltration, and inflammation [89]. To date, this cycle has not been described in cows, but, if exists, could offer a novel explanation of prolonged, severe lipolysis in the transition period.

ATM exhibiting the M2 phenotype possess a restorative and protective function, in contrast to the pro-inflammatory M1 type [90]. The main immune cell-derived inducers of non-classical macrophages are interleukins 4, 10, and 13 [91]. In human and mouse studies, the alternative macrophage phenotype has been induced by omega-3 FA molecules and pharmacological agents through peroxisome proliferator-activated receptors [92–94]. Given the intense ATM polarization to M1 during excessive lipolysis in the transition period, the possibility of modulating ATM phenotype in transition dairy cows may offer therapeutic alternatives [81].

Reports on trafficking and inflammatory responses of adipose immune cells, other than ATM, in dairy cows are limited. T-cells were reported to comprise 3–5% of the SVF cells in cows, with or without displaced abomasum, during the transition period and in late lactation [81, 82, 95]. Around 4–7% of SVF cells are B lymphocytes and their numbers are higher in visceral AT than other AT. Interactions among different types of immune cells, adipocytes, and other cells in the SVF of AT modulate metabolic responses during the transition period. Therefore, a better understanding of immune cell population trafficking and inflammatory responses is necessary to develop strategies to modulate AT metabolism, especially lipolysis.

### Cell proliferation

AT is a plastic organ that adapts to metabolic challenges by expansion or contraction of adipocyte size and numbers. Furthermore, adipocytes have a constant turnover that requires recruitment of new progenitors regularly [96]. Lipolysis induces the recruitment of new adipocytes in rodent models of obesity and adrenergic signaling [97]. In dairy cows, assessment of cell turnover is difficult because genetic tracing tools are not available for large animals. Markers of cell cycle activation, such as *CCNA2*, *CCNB2*, and *MKI67* have been used. In late-lactation cows, short-term intense lipolysis did not increase the expression of these genes [82]. In dairy heifers during peak lactation, Häussler et al. detected a 25-fold increase in the number of preadipocytes in the retroperitoneal AT [98], compared to heifers in mid lactation (105 DIM), using immunohistochemistry with antibodies against Ki67, a cell proliferation marker, and Pref-1 (preadipocyte factor). Remarkably, visceral AT apoptosis was also increased in early lactation compared to mid lactation, as demonstrated by the Terminal deoxynucleotidyl transferase dUTP nick end labeling (TUNEL) assay that detects DNA fragments as a measure of apoptosis. The implications of a potentially increased rate of cell proliferation in AT during the transition period is currently unknown. Increased preadipocyte proliferation, and therefore adipogenesis, especially in subcutaneous AT, would likely be beneficial to transition cow metabolism, by, for example, improving the AT capacity to buffer excess FA released during lipolysis, as has been shown in humans and rodent models of insulin signaling dysregulation [99]

### Extracellular matrix

The ECM of AT gives structural support to its cellular components. Proteoglycans and fibrous proteins, such as collagens, are the main classes of AT ECM proteins, and their functions and distribution were recently reviewed in detail by Mariman and Wang [100]. In human obesity, characterized by excessive rates of basal lipolysis, there is enhanced deposition of collagens I and VI and thrombospondin-1 [101–103]. The presence of these ECM proteins is also associated with ATM infiltration and polarization to the M1 phenotype [102, 104]. High rates of lipolysis observed in cancer cachexia are also associated with changes in the ECM composition that lead to enhanced inflammatory responses by adipocytes [105, 106]. In late-lactation dairy cows, ECM composition is affected by anatomical location, with higher expression of collagens I and VI in subcutaneous fat compared to omental fat [82]. Reports on the dynamics of ECM remodeling during the transition period and early lactation are limited to gene expression evaluation. Akbar et al., [107] reported higher expression of collagen I in subcutaneous AT of transition cows with metritis, compared to healthy animals. In feed-deprived lactating goats, Faulconnier and colleagues described enhanced transcription of *COL3A1* [84]. A reanalysis of microarray data by Sumner-Thompson et al., [108] looking for enrichment in different ECM-related groupings via the Database for Annotation, Visualization, and Integrated Discovery revealed that *THBS1* expression is upregulated during peak lipolysis at the onset of lactation. Proteome analysis focused on the transition period is necessary to evaluate if lipolytic responses during the transition period induce changes in the composition of ECM that impair AT function.

## Impact of AT lipolysis and remodeling

Dysregulated inflammatory responses and oxidative stress are recognized as key components of the metabolic stress syndrome in transition dairy cows [7, 109]. AT remodeling and lipolysis can be considered the third key component, also leading to metabolic stress through two different mechanisms. First, and as demonstrated initially in non-ruminants, lipolysis modulates the phenotype of immune cells [110]. FA and other lipolytic products are potent activators of inflammatory pathways in mononuclear cells, lymphocytes, and polymorphonuclear cells [48, 111]. In dairy cows, FA impair the immune responses of lymphocytes and neutrophils by enhancing the pro-inflammatory response and simultaneously reduce their capacity to clear pathogens [64, 112]. Second, excessive lipolysis is a trigger of oxidative stress. In monogastrics, FA in circulation and in tissues become oxidized by free radicals and rapidly deplete antioxidant reserves, leading to oxidative stress [101]. In dairy cows, specific oxidized products of arachidonic acid, 11-hydroxyeicosatetraenoic acid (11-HETE), and linoleic acid, 9-hydroxyoctadecadienoic acid (9-HODE) and 13-HODE, significantly increase during the transition period coinciding with enhanced lipolytic rate [113]. Modulating the rate and intensity of FA release from AT through nutritional interventions, including supplementation of methyl donors choline and methionine [114], and ω-6 polyunsaturated FA [104, 115, 116], improves immune function, reduces oxidative stress, and enhances adipose insulin sensitivity. These studies further demonstrate the importance of lipolysis as a determinant factor for disease susceptibility in transition dairy cows.

Excessive lipolysis and AT remodeling may also impair lactation performance. Increased concentrations of lipolysis biomarkers NEFA and β-hydroxybutyrate during the transition period are associated with reduced milk production and impaired reproductive function [117]. In monogastrics, AT inflammation, a feature of AT remodeling, is directly linked with the development of AT-specific insulin resistance (IR) [11]. In transition cows a transient state of IR is considered homeorhetic, as it guarantees glucose supply for milk synthesis by limiting glucose use by peripheral tissues and triggering AT lipolysis [12]. However, extended periods of IR during the transition period may increase and prolong AT lipolysis, as observed in women with gestational diabetes and metabolic syndrome patients [118, 119]. During active AT remodeling, the expression and secretion of IL1-β, IL-6, resistin, and TNF-α by human adipocytes and ATM is increased, leading to impaired AT insulin signaling [74, 88]. In transition dairy cows, AT expression of IL-6, resistin, TNF-α, and other bioactive peptides associated with IR is increased compared to other stages of lactation [120–122]. Recent studies provide evidence for AT-specific IR during early lactation in high-yielding dairy cows. Zachut et. al [38] demonstrated a significant reduction in the phosphorylation of downstream insulin signaling pathways, such as IRS-1 and AKT, in subcutaneous adipose, while the activation of these pathways remained intact in the liver. Remarkably, adipose-specific IR was only observed in cows that had higher rates of lipolysis and lost more body condition during early lactation. Despite this evidence, the role of periparturient AT remodeling in the development of IR is still a matter of debate, as Mann and colleagues did not observe any changes in insulin signaling pathways in over-conditioned cows with excessive lipolytic rates [123]. Furthermore, throughout lactation the degree of IR is dynamic and was shown to increase during late lactation, a period characterized by reduced lipolytic activity [124]. These reports underscore that lipolysis regulation by insulin signaling and AT remodeling is a complex process that requires further characterization.

## Conclusions

**Fig. 1:**
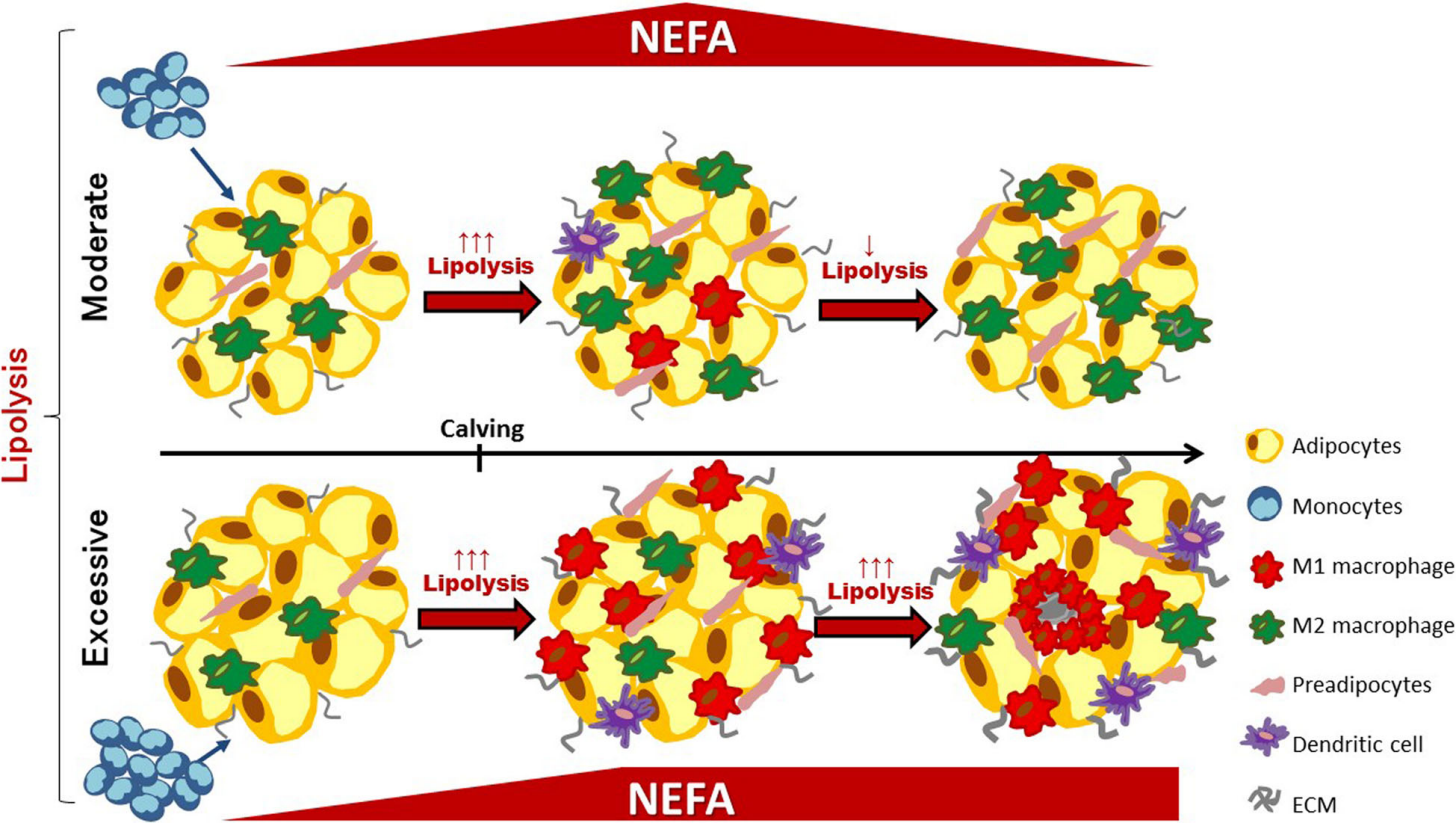
Lipolysis induces adipose tissue (AT) remodeling. This process is characterized by macrophage infiltration and changes in inflammatory phenotype. During moderate lipolysis, macrophage infiltration is limited and involves mainly the M2 phenotype (anti-inflammatory). In contrast, during excessive lipolysis, most infiltrating macrophages are M1 (pro-inflammatory) and enhance lipolysis and reduce adipocyte insulin sensitivity. Excessive lipolysis and AT remodeling increase disease susceptibility and negatively impact lactation performance

The impact of intense and extended periods of AT lipolysis on transition cow disease susceptibility and lactation performance may be directly related to the remodeling process, alterations in the expression of adipokines, and the development of insulin resistance (Fig. 1). Certainly, AT biology research focus on human obesity and the diabetes epidemic in western countries has expanded our understanding of the role of lipolysis in metabolic and immune function. However, further research on AT lipolysis and remodeling is required to elucidate specific inflammatory and metabolic pathways that link adipocyte and immune cell function in dairy cattle. Improving our understanding of AT function in transition dairy cattle will lead to the identification of new biomarkers of disease and productivity, which will allow for improved herd health and profitability.

## Abbreviations

AT: Adipose tissue
ATM: Adipose tissue macrophages
FA: Fatty acids
M1: Pro-inflammatory phenotype
M2: Anti-inflammatory phenotype
NEFA: Non-esterified fatty acids
TAG: Triacylglycerols
